# An anomalous ferroelastic phase transition arising from an unusual *cis*-/*anti*-conformational reversal of polar organic cations†

**DOI:** 10.1039/d3sc01101a

**Published:** 2023-05-02

**Authors:** Bing-Qing Zhao, Xiao-Xian Chen, Hui Ye, Ya-Ping Gong, Jun Wang, Le Ye, Wei-Xiong Zhang

**Affiliations:** a MOE Key Laboratory of Bioinorganic and Synthetic Chemistry, School of Chemistry, Sun Yat-Sen University Guangzhou 510275 China zhangwx6@mail.sysu.edu.cn

## Abstract

Hybrid ferroelastics have attracted increasing attention for their potential application as mechanical switches. The sporadically documented anomalous ferroelastic phase transitions, *i.e.*, ferroelasticity that appears at a high-temperature phase rather than a low-temperature phase, are of particular interest but are not well understood at the molecular level. By judiciously choosing a polar and flexible organic cation (Me_2_NH(CH_2_)_2_Br^+^) with *cis*-/*anti*- conformations as an A-site component, we obtained two new polar hybrid ferroelastics, A_2_[MBr_6_] (M = Te for 1 and Sn for 2). These materials undergo distinct thermal-induced ferroelastic phase transitions. The larger [TeBr_6_]^2−^ anions anchor the adjacent organic cations well and essentially endow 1 with a conventional ferroelastic transition (*P*2_1_ → *Pm*2_1_*n*) arising from a common order-disorder transition of organic cations without conformational changes. Moreover, the smaller [SnBr_6_]^2−^ anions can interact with the adjacent organic cations in energetically similar sets of intermolecular interactions, enabling 2 to undergo an anomalous ferroelastic phase transition (*P*2_1_2_1_2_1_ → *P*2_1_) arising from an unusual *cis*-/*anti*-conformational reversal of organic cations. These two instances demonstrate the importance of the delicate balance of intermolecular interactions for inducing anomalous ferroelastic phase transitions. The findings here provide important insights for seeking new multifunctional ferroelastic materials.

## Abstract 2

Two polar hybrid ferroelastic materials with the same organic cations, but slightly different inorganic octahedra, undergo ferroelastic phase transitions whereby the distinct symmetry breaking essentially arose from conformations of organic cations.

## Graphical abstract

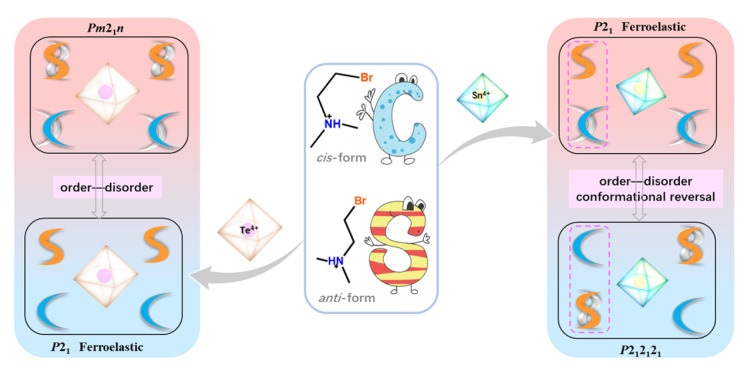

## Introduction

Ferroelasticity is a mechanical analog of ferromagnetism and ferroelectricity, which offers the unique mechanical properties of spontaneous strain, strain–stress hysteresis, and domain conversion. Extensive research on metal alloys and ceramics has revealed the important roles of ferroelasticity in mechanical switches and piezoelectric sensors.^1–8^ Traditionally, displacive-type inorganic ferroelastics, such as Gd_2_(MoO_4_)_3_ and Pb_3_(PO_4_)_2_, require solid-state synthesis at high temperatures.^9,10^ Hybrid ferroelastics, as important supplements, could be assembled in solutions under moderate conditions from diverse organic and inorganic components, hence offering an eco-friendly and feasible approach for designing ferroelastic materials.^11–14^ Recent years have witnessed the emergence of numerous hybrid ferroelastics,^15–27^ such as (Me_3_NOH)_2_[ZnCl_4_] with a large spontaneous strain of 0.129,^28^ (nortropinium)[CdCl_3_] with the ultrahigh ferroelastic phase-transition temperatures of 463 and 495 K,^29^ and the chiral (*R*-3-chloro-2-hydroxypropyltrimethylammonium)_2_CuCl_4_ with seven physical channel switches.^30^

According to tests on the 94 species of ferroelastic crystals suggested by Aizu, decreasing temperatures favor a symmetry reduction, and ferroelasticity generally appears at the low-temperature phase (LTP) during a conventional ferroelastic phase transition.^31,32^ However, due to the complexity arising from the weak but abundant intermolecular interactions in molecule-based materials, some anomalous cases may occur, *i.e.*, ferroelasticity appears at the high-temperature phase (HTP) rather than LTP.^33,34^ One well-known example is Rochelle salt (KNaC_4_H_4_O_6_·4H_2_O),^35^ which undergoes a phase transition at 255 K from paraelastic LTP in an orthogonal space group, *P*2_1_2_1_2 (point group 222), to ferroelastic HTP in the monoclinic space group, *P*2_1_ (point group 2). In 2021, a molecular rotor crystal, [Zn(saloph)]_2_(μ-dabco) (H_2_saloph = *N*,*N*′-bis(3,5-di-*tert*-butylsalicylidene)-1,2-phenylenediamine, dabco = 1,4-diazabicyclo[2.2.2]octane),^36^ was found undergoing an anomalous ferroelastic transition at 263 K from paraelectric LTP (*Cmca*) to ferroelastic HTP (*P*2_1_/*c*); in this material the unequal motions of the rotators and anisotropic steric repulsion between the molecules make an important contribution to transition. Recently, we found that an anti-XeF_4_ molecular analog, (Me_3_NCH_2_CH_2_OH)_4_[Ni(NCS)_6_],^37^ shows an unprecedented high- and low-temperature dual ferroelasticity with point-group changes of 2/*m*–4/*mmm*–*mmm*; in this material the anomalous ferroelastic HTP is strongly associated with the synergistic swaying of both organic and inorganic components through intermolecular hydrogen bonds. Understanding the origins of anomalous ferroelastic transitions at the molecular level in hybrid crystals is of particular importance for designing advanced high-temperature hybrid ferroelastics as it allows combining multiple functionalities, such as polar or chiral structures. However, among the sporadically documented anomalous ferroelastic transitions, only very rare cases involve polar structures.^35^ Therefore, it remains a challenge to design anomalous ferroelastic transitions, owing to the scarcity of instances and the complexity of the molecular components.

**Scheme 1:**
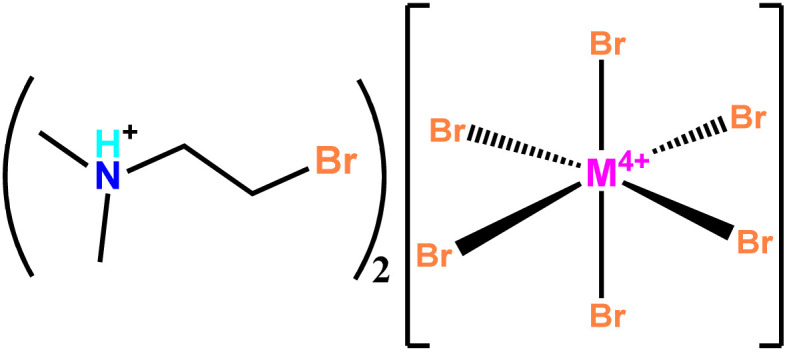
The structural formulae of 1 and 2 (M refers to either Te or Sn, respectively).

In our ongoing studies on phase-transition hybrid crystals assembled by polar organic cations and inorganic components,^11–13^ we found some organic cations with changeable *cis*-/*anti*- conformations that can serve as unique components to induce polar structures, such as Me_3_N(CH_2_)_2_Br^+^. These polar structures bring about ferroelectric transition by changing conformations in the first hybrid postperovskite ferroelectric.^38^ Considering that the discrete inorganic octahedra facilitate a more complicated and delicate interplay between the inorganic and organic components, we assembled Me_2_NH(CH_2_)_2_Br^+^ with diverse discrete inorganic coordination octahedra to seek more uncommon phase transitions. Our efforts yielded two new polar hybrid crystals, (Me_2_NH(CH_2_)_2_Br)_2_[MBr_6_] (M = Te for 1, and Sn for 2, Scheme 1). We comprehensively investigated their structural phase transitions using thermal analyses, *in situ* variable-temperature single-crystal/powder X-ray diffraction, dielectric, and second-harmonic generation (SHG) measurements. We found that the synthesized materials undergo thermally induced ferroelastic crystalline phase transitions and solid–liquid transitions accompanied by similar thermally induced “low-high-low” SHG-switching behaviors. However, the slightly changed inorganic octahedra bring about distinct ferroelastic phase transitions, *i.e.*, a conventional ferroelastic transition (*P*2_1_ → *Pm*2_1_*n*) associated with a common order-disorder transition of organic cations for 1 and an anomalous ferroelastic transition (*P*2_1_2_1_2_1_ → *P*2_1_) associated with an unusual *cis*-/*anti*-conformational reversal of organic cations for 2. The underlying delicate balance of the intermolecular interactions for such distinct ferroelastic transitions was discussed *via* Hirshfeld surface analyses, which provides important clues for designing advanced hybrid ferroelastics.

## Experimental section

### Materials and instruments

All chemicals were commercially purchased and were used without further purification. Powder X-ray diffraction (PXRD) patterns were recorded using a Bruker D8 ADVANCE X-ray powder diffractometer (Cu K_α_, *λ* = 1.54184 Å). Thermogravimetric analyses (TGA) were performed using a TA Q50 system at a heating rate of 10 K min−^1^ under a nitrogen atmosphere. Differential scanning calorimetry (DSC) measurements were performed by heating and cooling the powdered sample at a rate of 10 K min^−1^ on a TA DSC Q2000 instrument. The dielectric measurements were carried out on a TH2838 impedance analyzer at frequencies from 5 kHz to 2.0 MHz, with an applied voltage of 1.0 V and a temperature sweeping rate of 3 K min^−1^ approximately in the range of 100–440 K in a Mercury iTC cryogenic environment controller of Oxford Instrument. The pressed-powder pellets were fixed by two magnetic sheets acting as electrodes. Variable-temperature polarization microscopy observations were carried out on a polarizing microscope OLYMPUS BX41-P equipped with a Linkam cooling/heating stage THMSE 600. The temperature was stabilized to an accuracy of ±0.1 K. The SHG effect was measured using an XPL1064-200 instrument at a heating/cooling rate of 2 K min^−1^.

### Synthesis of 1 and 2

The red block crystals of 1 and yellow plate crystals of 2 were obtained by slow evaporation of mixed HBr aqueous solutions of (Me_2_NH(CH_2_)_2_Br)Br and corresponding metal oxide (TeO_3_ and SnO for 1 and 2, respectively) in a molar ratio of 1 : 2 for several days at room temperature. The yields were 90% and 95% for 1 and 2, respectively. The phase purities of the poly-crystals were confirmed by PXRD analyses (Fig. S1, ESI†).

### X-ray crystallography of 1 and 2

The variable-temperature single-crystal X-ray diffraction intensities were collected on an Agilent SuperNova single-crystal diffractometer with graphite monochromated Cu K_α_ (*λ* = 1.54184 Å) radiation. Absorption corrections were applied by using the multi-scan program, CrysAlisPro. The structures were solved by the direct methods and refined by the full-matrix least-squares method with the SHELX program package on the Olex^2^ program.^39,40^ The hydrogen atoms were generated geometrically. All non-hydrogen atoms were refined using anisotropic thermal parameters. The positions of the hydrogen atoms were generated geometrically. Detailed crystallographic data and structural refinement parameters are listed in Table 1. The CCDC numbers were 2222445–2222446 for 1 and 2222447–2222448 for 2, respectively.

**Table 1:** Crystal data and structure refinement parameters for 1 and 2

Parameter	(C_4_H_11_NBr)_2_[TeBr_6_] (1)		(C_4_H_11_NBr)_2_[SnBr_6_] (2)	
Formula weight	913.08		904.24	
Phase	1_LTP	1_HTP	2_LTP	2_HTP
Temperature (K)	150(2)	325(2)	293(2)	350(2)
Crystal system	Monoclinic	Orthorhombic	Orthorhombic	Monoclinic
Space group	*P*2_1_	*Pm*2_1_*n*	*P*2_1_2_1_2_1_	*P*2_1_
*a*/Å	8.5323(5)	8.7336(8)	17.2270(2)	8.7521(7)
*b*/Å	9.6457(6)	9.8400(8)	9.6868(1)	9.7104(6)
*c*/Å	13.8826(8)	13.801(1)	14.0647(3)	14.0201(1)
*β*/^o^	95.252(3)	90	90	94.430(8)
*V*/Å^3^	1137.7(2)	1186.1(2)	2347.04(5)	1188.0(2)
*Z*	2	2	4	2
*D*_c_/(g cm^−3^)	2.665	2.557	2.559	2.528
*R*_1_ [*I* > 2σ(*I*)]^a^	0.0586	0.0540	0.0406	0.0796
*wR*_2_ [*I* > 2*σ*(*I*)]^b^	0.1714	0.0823	0.1121	0.2553
GOF	1.085	0.993	1.054	1.066
Flack parameter	/c	0.02(3)	−0.001(6)	0.01(2)
CCDC number	2222445	2222446	2222447	2222448

a *R*_1_ = ∑‖*F*_o_| − |*F*_c_‖/∑|*F*_o_|.

b *wR*_2_ = [∑*w*(*F*_o_^2^ − *F*_c_^2^)^2^/∑*w*(*F*_o_^2^)^2^]^1/2^.

c For 1_LTP: TWIN LAW (1 0 0 0 1 0 0.298 0 −1), BASF [0.016(2)].

## Results and discussion

### Thermal analyses

**Fig. 1:**
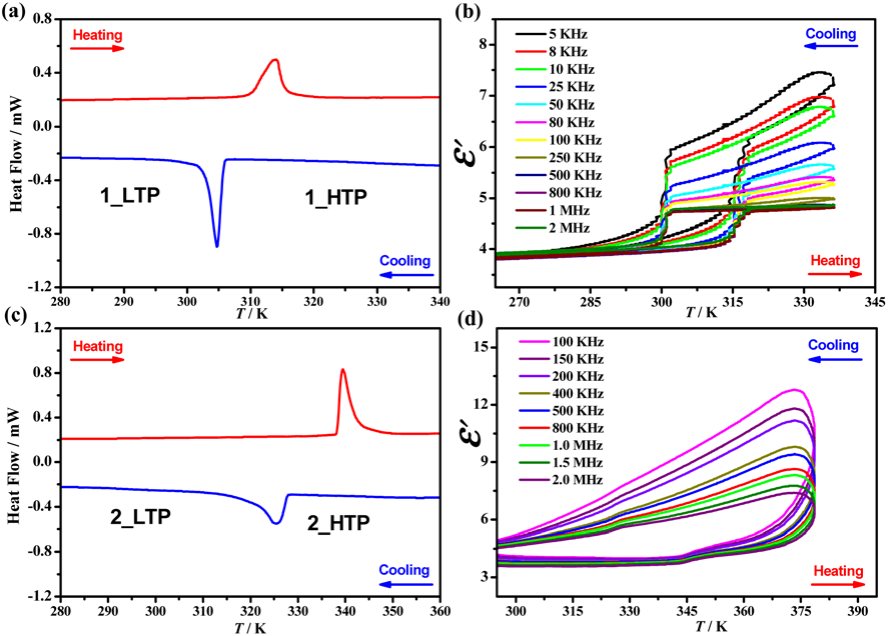
DSC curves for 1 (a) and 2 (c). Dielectric constants were measured at different frequencies in a heating–cooling cycle for 1 (b) and 2 (d).

TGA measurements showed that 1 and 2 could be stable up to 453 K (Fig. S2†) under the N_2_ atmosphere. DSC tests in the temperature range of 190–435 K revealed that 1 and 2 undergo reversible solid–liquid phase transitions at similar melting/solidification temperatures of 404/371 K and 399/373 K, respectively (Fig. S3†). In addition, as shown in Fig. 1 and S4,† the DSC tests at temperatures as low as 100 K detected a pair of endothermic/exothermic thermal anomalies at 314/306 K and 340/325 K in the heating/cooling runs, with thermal hystereses of 8 and 15 K for 1 and 2, respectively, indicating reversible one-step crystalline phase transitions; this was further confirmed by variable-temperature PXRD patterns (Fig. S5 and S6†). In the heating/cooling runs, the enthalpy changes (Δ*H*) and entropy changes (Δ*S*) during the crystalline phase transition in 1 were 2.6/3.4 kJ mol^−1^ and 8.6/11.0 J mol^−1^ K^−1^, respectively, both are higher than those observed in 2 (1.3/1.2 kJ mol^−1^ and 4.0/3.6 J mol^−1^ K^−1^, respectively), implying a more significant change in molecular dynamics for 1 (*vide infra*). For convenience, the phases of 1 below and above 314 K are labeled as 1_LTP and 1_HTP, and the phases of 2 below and above 340 K are labeled as 2_LTP and 2_HTP.

### Temperature-dependent dielectric responses

Such reversible crystalline phase transitions were further confirmed by a temperature-dependent dielectric constant (*ε*′, Fig. 1b and d). For example, the *ε*′ measured at a frequency of 2 MHz (Fig. S7†) for 1 exhibits a steep increase from *ca.* 3.9 at 265 K (1_LTP) to *ca.* 4.9 at 335 K (1_HTP) in a heating run; a reverse drop then occurs at ∼301 K in the cooling run that follows. In contrast, for 2, *ε*′ exhibits a slight jump from *ca.* 3.6 at 295 K (2_LTP) to *ca.* 4.1 at 355 K (2_HTP), maintains a gradual increase upon heating, and then exhibits a reverse gradual decrease in the cooling run that follows. Such different temperature dependence of *ε*′ values implies that the changes in the molecular dynamics of polar organic cations during the phase transition in 1 are much brisker than those in 2.

### Structural analyses

**Fig. 2:**
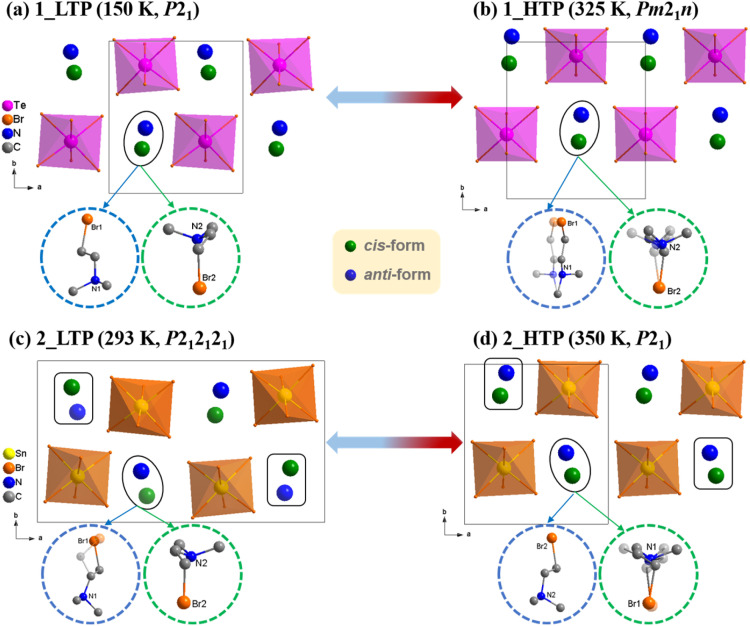
Crystal packing structures in 1_LTP (a), 1_HTP (b), 2_LTP (c), and 2_HTP (d). For clarity, H atoms are omitted and the *cis*-form and *anti*-form conformations of the (Me_2_NH(CH_2_)_2_Br^+^) cations are represented as green and blue balls, respectively.

To investigate the structural phase transitions, *in situ* variable-temperature single-crystal X-ray diffraction measurements were performed for 1 and 2, respectively. As illustrated in Fig. 2, both 1 and 2 could be roughly described as a polar A_2_MX_6_-type structure, in which each M^IV^ ion is coordinated with six Br^−^ ions to form discrete [M^IV^Br_6_]^2−^ octahedra separated by A-site (Me_2_NH(CH_2_)_2_Br^+^) cations with *cis*- and *anti*- conformations in 1 : 1 ratio. It should be noted that 1_LTP and 2_HTP crystallize in the same polar space group, *P*2_1_ (No. 4), with similar cell parameters. However, 1 and 2 exhibit distinct symmetry-breaking phase transitions upon heating, *i.e.*, *P*2_1_ → *Pm*2_1_*n* for 1 and *P*2_1_2_1_2_1_ → *P*2_1_ for 2, respectively (Table 1).

In detail, 1_LTP belongs to a monoclinic space group (*P*2_1_, No. 4) with cell parameters of *a* = 8.5323(5) Å, *b* = 9.6457(6) Å, *c* = 13.8826(8) Å, *β* = 95.252(3)°, and *V* = 1137.74(12) Å^3^. Its asymmetric unit contains a complete [TeBr_6_]^2−^ octahedron and two crystallographically ordered organic cations in *cis*- and *anti*-conformations, respectively (Fig. S12a†). 1_HTP belongs to an orthorhombic space group (*Pm*2_1_*n*, No. 31), in which the asymmetric unit becomes halved because of the additional crystallographic mirror symmetry (Fig. S12b†); in addition, each organic cation maintains its conformation but becomes 2-fold disordered about the mirror plane perpendicular to the *a*-axis (Fig. S13†). In short, the reversible 1_LTP ↔ 1_HTP phase transition can mainly be ascribed to the order-disorder transitions of organic cations, and it obeys a group–supergroup relationship with a normal symmetry-breaking change.

At 350 K (2_HTP), 2 presented a similar structure as 1_LTP, except the *cis*-conformational organic cation was crystallographically 2-fold disordered on general position with site occupancies of 0.42 and 0.58 (Fig. S11d†). Upon cooling to 2_LTP, the space group changed from monoclinic *P*2_1_ into orthorhombic *P*2_1_2_1_2_1_ (No. 19) with the *Z* values varying from 2 to 4. In comparison with the 2_HTP, 2_LTP had a doubled *a*-axis (8.7521(7) Å *vs.* 17.2270(2) Å) as half of the organic cations reverse their conformations, while slightly changing positions to yield an additional 2_1_ screw axis along the *a* axis. Moreover, for the two crystallographically dependent organic cations, the *anti*-form one was crystallographically ordered in 2_HTP but became 2-fold disordered on general position with the site occupancies of 0.48 and 0.52 in 2_LTP. Contrarily, the *cis*-form one being disordered in 2_HTP became ordered in 2_LTP. In short, differing from 1, 2 underwent a much more complicated crystalline phase transition that obeyed an unusual group–supergroup relationship, *i.e.*, orthorhombic LTP to monoclinic HTP, which was accompanied by an uncommon *cis*-/*anti*-conformational reversal of flexible organic cations.

### Temperature-dependent lattice parameters

**Fig. 3:**
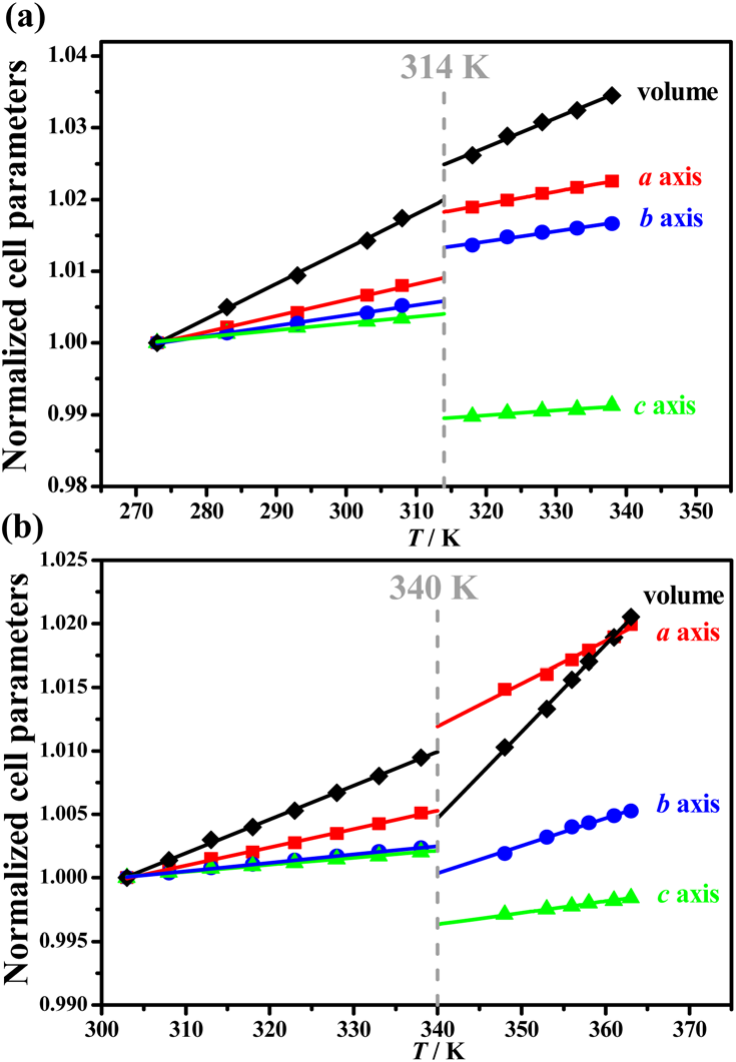
Temperature dependence of normalized cell parameters for 1 (a) and 2 (b).

Such distinct phase transitions in 1 and 2 were also reflected in their differences in the temperature dependence of their cell parameters. Pawley refinements were performed on variable-temperature PXRD patterns to obtain the temperature-dependent lattice parameters (Tables S1–S4†) at 273–338 K for 1 and at 303–363 K for 2. The PASCal program^41^ was then employed to calculate the thermal expansion coefficients for each phase (Tables S5 and S6†). The volume expansion coefficient slightly decreased from +488(8) × 10^−6^ K^−1^ (1_LTP) to +394(12) × 10^−6^ K^−1^ (1_HTP) for 1, but significantly increased from +267(2) × 10^−6^ K^−1^ (2_LTP) to +678(13) × 10^−6^ K^−1^ (2_HTP)for 2. During the transition from 1_LTP to 1_HTP at 314 K, the lengths of the *a*-axis and *b*-axis increased by 0.92% and 0.75%, respectively, and a decrease of 1.4% was recorded in the *c*-axis length, together with a slight volume expansion by 0.48% (Fig. 3a). In contrast, during the transition from 2_LTP to 2_HTP at 340 K, the *a*-axis length increased by 0.67%, the *b*-axis and *c*-axis lengths decreased by 0.21% and 0.58%, respectively, together with an obvious volume shrinkage by about 0.52% (Fig. 3b). Such an abnormal volume shrinkage phenomenon during the 2_LTP → 2_HTP transition should be associated with the aforementioned *cis*-/*anti*-conformational reversal of the organic cations.

### Ferroelastic phase transitions and spontaneous strains

**Fig. 4:**
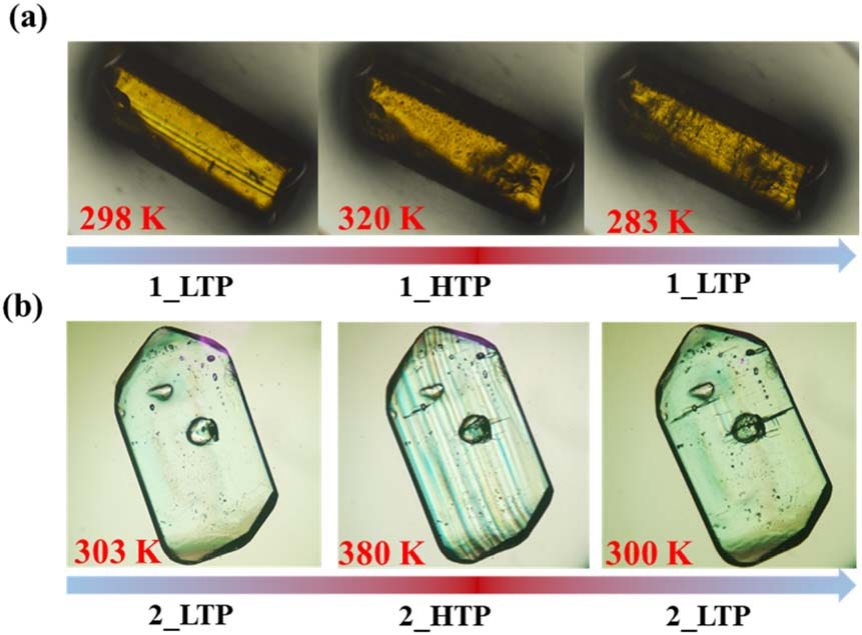
Variation in the multi-domain structures in a heating–cooling cycle for 1 (a) and 2 (b).

As indicated by the above structural analyses, during the phase transition, *Pm*2_1_*n* (1_HTP) → *P*2_1_ (1_LTP) in 1 and *P*2_1_2_1_2_1_ (2_LTP) → *P*2_1_ (2_HTP) in 2, the number of symmetry elements decreases from 4 (*E*, *C*_2_, and 2*σ*_v_) to 2 (*E* and *C*_2_) for 1 and from 4 (*E*, *C*_2_, and 
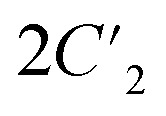
) to 2 (*E* and *C*_2_) for 2. These transitions belong to ferroelastic transitions with Aizu notation of *mm*2*F*2 and 222*F*2, respectively.^31^ To inspect these ferroelastic transitions, the variable-temperature polarization microscopy observations were performed on the single crystals of 1 and 2 by viewing them perpendicular to the (011) and (101) plane, respectively (Fig. S10 and S11†). As shown in Fig. 4, for 1, in the temperature range of 283–320 K, a stripe pattern could be distinguished on the crystal surface at 298 K, indicating the multiple ferroelastic domains with anti-parallel orientations for 1_LTP. These striped domains fade out when the crystal is heated to 320 K, indicating a paraelastic state for 1_HTP, and it could be recovered by cooling back to 283 K, indicating a recovered ferroelastic state for 1_LTP. Such a domain revolution strongly verified the reversible and normal ferroelastic phase transition for 1, *i.e.*, between a ferroelastic LTP and a paraelastic HTP, which is similar to the behaviors of most known ferroelastic materials.^17–29^ In contrast, in the temperature range of 300–380 K for 2, no multi-domain structure was observed at 303 K as it was in its paraelastic phase (2_LTP). Upon further heating to 380 K (2_HTP), the theoretically-predicted multiple domains with two anti-parallel orientation states could be clearly distinguished, which then completely disappeared after cooling back to 300 K. Therefore, as compared to 1 and most other ferroelastic materials, 2 is a rare ferroelastic material with a ferroelastic phase at HTP rather than at LTP.

Based on the cell parameters of 1 and 2 at 314 K and 340 K deduced by extrapolating the fitting lines of variable-temperature cell parameters, the total spontaneous strains were estimated as 0.059 and 0.062 for 1 and 2, respectively (for details, see ESI†).^32,42^ These spontaneous strain values are of intermediate level for other hybrid ferroelastics,^15–29^ but the value for 2 (0.062) was much larger than those observed in the documented anomalous ferroelastics with inverse symmetry breaking, such as [Zn(saloph)]_2_(μ-dabco) (0.02),^36^ (Me_3_NCH_2_CH_2_OH)_4_[Ni(NCS)_6_] (0.0073),^37^ and (Et_4_N)(Me_4_N)[MnBr_4_] (0.0043).^43,44^ These results implied that the anomalous ferroelastic transition and large spontaneous strain in 2 should be strongly associated with not only the order-disorder dynamic transition of cations but also the *cis*-/*anti*-conformational variations in organic cations.

### Temperature-dependent SHG responses

**Fig. 5:**
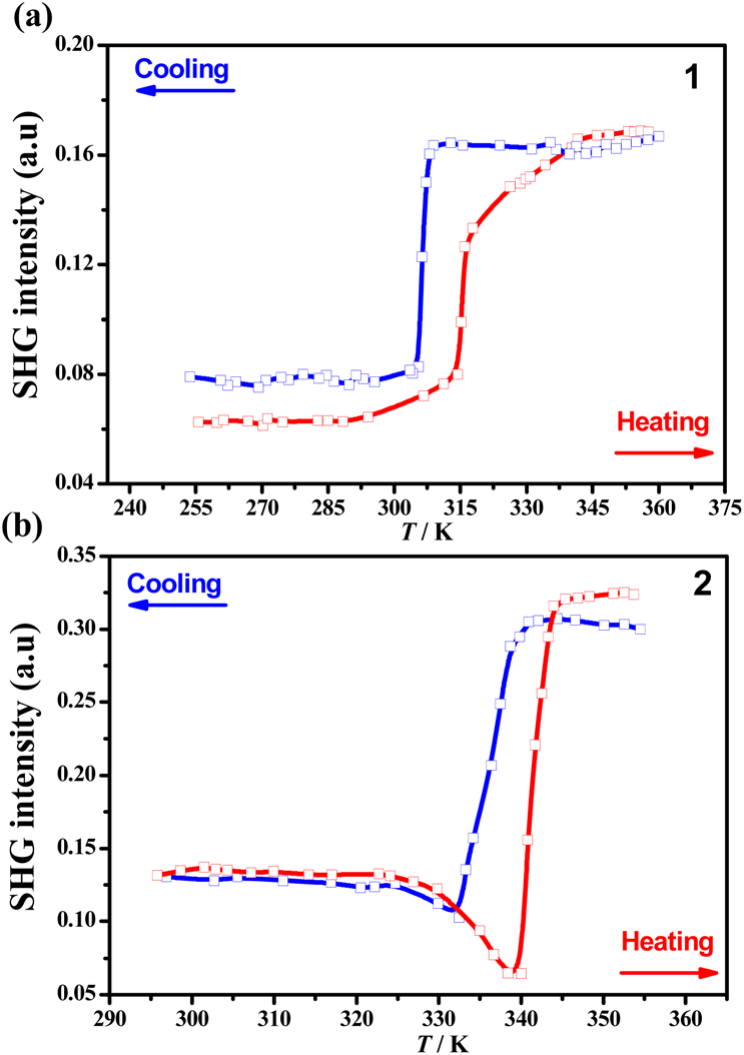
Temperature dependence of normalized cell parameters for 1 (a) and 2 (b).

The temperature-dependent SHG responses, which are sensitive to the inversion symmetry breaking,^45–47^ were recorded by applying the Kurtz and Perry model to the polycrystalline samples (*λ* = 1064 nm, Nd:YAG pulsed laser)^48^ of 1 and 2. The SHG intensity at room temperature (Fig. 5) was about 0.06 and 0.13 times that of potassium dihydrogen phosphate (KDP) for 1_LTP and 2_LTP, respectively, and increased to 0.16 and 0.32 times that of KDP for 1_HTP and 2_HTP, respectively; it eventually dropped to 0.03 and 0.02 times that of KDP for molten 1 and 2, respectively (Fig. S8b and S9b†).^49^ Such two-step “low-high-low” SHG-switching behaviors during one-step crystalline phase transition and one-step solid–liquid transition with a rather high switching contrast of 2.6 and 5.3 for 1, 2.5 and 16 for 2, respectively, have rarely been observed. They differ from most of the known SHG switches that present only a one-step “high-low” SHG switching upon heating.^50–53^ As the inorganic anions are almost non-polar, the SHG intensification observed in the HTPs should be mainly ascribed to the polar arrangements and the molecular dynamics of the polar organic cations (Me_2_NH(CH_2_)_2_Br^+^) with a terminal heavy bromine atom. In detail, these crystallographically disordered polar organic cations in the HTP phase make their dipole moment superimpose along the polarization direction (*i.e.*, the *b*-axis), hence enhancing the molecular polarization and then the SHG signals. This observation is consistent with the theoretical calculation of the polarization intensity according to the point charge model^54,55^ along the polarization direction for 1 and 2, which revealed that both 1_HTP and 2_HTP possess larger polarization than the 1_LTP and 2_LTP, respectively (Table S9†). It should be noted that, differing from the case in 1, the SHG signal did not monotonically increase during the 2_LTP → 2_HTP transition but temporarily decreased in the vicinity of the phase transition (Fig. 5b).^56–58^ Such an abnormal temporary decrease in the SHG response should be strongly associated with the *cis*-/*anti*-conformational reversal process of the organic cations, during which the polar arrangement of the organic cations may be temporarily destroyed to a certain degree.

### Hirshfeld surface analyses

**Fig. 6:**
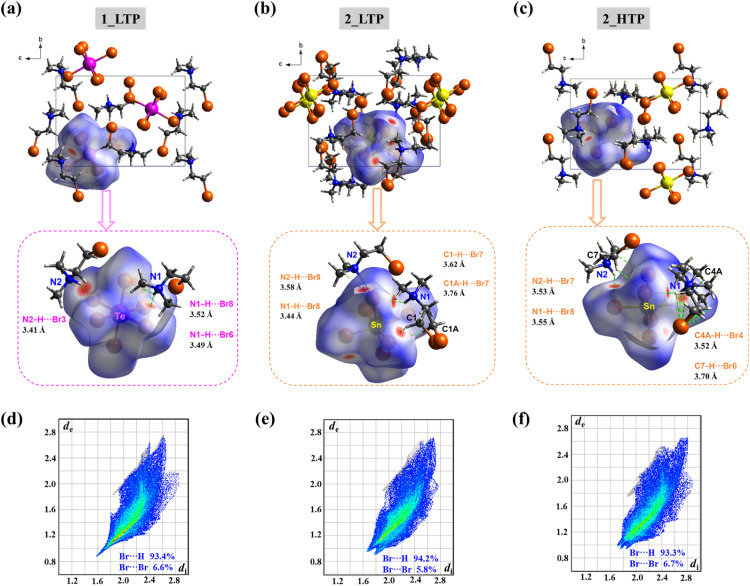
The Hirshfeld surfaces and fingerprint plots for [TeBr_6_]^2−^ and [SnBr_6_]^2−^ in 1_LTP (a and d), 2_LTP (b and e), and 2_HTP (c and f), respectively. Inset: Hirshfeld surfaces show stronger short contacts (red spots) with neighboring atoms.

Considering that 1 and 2 have the same organic cations (Me_2_NH(CH_2_)_2_Br^+^) but slightly different inorganic octahedra, it is reasonable to claim that the subtle change in metal ions makes an essential contribution to the distinct phase transitions of 1 and 2. This happens by changes in the conformations and orientations of organic cations as well as the intermolecular interactions involved. As shown by the DSC measurements, the melting points of 1 and 2 were almost the same, implying that they had similar intermolecular interactions between cations and anions. To find out the detail of the differences in their intermolecular interactions, Hirshfeld surfaces^59^ were calculated for [TeBr_6_]^2−^ in 1_LTP, [SnBr_6_]^2−^ in 2_LTP and 2_HTP, respectively. As shown in the decomposed fingerprint plots (Fig. 6d and e), approximately 93.4% and 94.2% of surface area were associated with the Br⋯H–N/C interactions for 1_LTP and 2_LTP, respectively. This made major contributions to the attractive intermolecular interactions between the inorganic octahedron and organic cations, while the rest of the surface areas associated with the Br⋯Br weak interactions between the coordinated Br atom in the inorganic octahedra and the terminal Br atom in the organic cation made only minor contributions. Such similar percentages further confirmed the similarity of inorganic–organic intermolecular interactions in 1 and 2.

However, because of the larger ionic radius of Te^IV^ (97 pm) than Sn^IV^ (69 pm),^60^ the [TeBr_6_]^2−^ octahedron has a larger size than [SnBr_6_]^2−^, as reflected by the longer Te–Br bonds (2.670(2)–2.736(2) Å) than the Sn–Br ones (2.581(1)–2.619(1) Å) (Tables S7 and S8†).^61^ For the same number of organic cations to interact, the smaller [SnBr_6_]^2−^ anion was more crowded than [TeBr_6_]^2−^ anion, and thus, brought about slightly different intermolecular interactions in 1 and 2. As indicated by the red spots on the Hirshfeld surface of [TeBr_6_]^2−^ in 1_LTP, besides the major strong inorganic–organic interactions, *i.e.*, the N–H⋯Br (*d*_N⋯Br_ = 3.41–3.52 Å) weak hydrogen bonds, some weak C–H⋯Br and C–Br⋯Br (*d*_Br⋯Br_ = 3.68–4.04 Å) interactions were formed to complement the intermolecular interactions for both inorganic anions and organic cations. Namely, the larger [TeBr_6_]^2−^ anions anchored with the adjacent organic cations to be in a crystallographically ordered state in 1_LTP; it eventually yielded a one-step order-disorder 1_LTP → 1_HTP transition by swaying the organic cations along their long axes but maintaining their *cis*-/*anti*-conformations.

By contrast, the smaller [SnBr_6_]^2−^ anion was too crowded to satisfy all the interactions for the adjacent organic cations, bringing about more possible local structures with different but very similar intermolecular interactions. Accordingly, the organic cations in both 2_LTP and 2_HTP were neither crystallographically ordered (the case in 1_LTP), nor crystallographically disordered (the case in 1_HTP), but were only partially crystallographically disordered. The major attractive intermolecular interactions (Fig. 6d and f), *i.e.*, N–H⋯Br and C–H⋯Br interactions, in 2_LTP and 2_HTP, respectively, were similar in general but presented the relation of “as one falls, another rises”. In detail, as indicated by the red spots on the Hirshfeld surfaces of [SnBr_6_]^2−^ anions (Fig. 6c and e), the N–H⋯Br interactions in 2_LTP (*d*_N⋯Br_ = 3.44–3.58 Å) are stronger than those in 2_HTP (*d*_N⋯Br_ = 3.53–3.55 Å), while the C–H⋯Br ones in 2_LTP (*d*_C⋯Br_ = 3.62–3.76 Å) are weaker than those in 2_HTP (*d*_C⋯Br_ = 3.52–3.70 Å). In addition, the relatively longer Br⋯Br distance in 2_LTP (*d*_Br⋯Br_ = 3.81–4.07 Å) and 2_HTP (*d*_Br⋯Br_ = 3.84–4.15 Å) than that in 1_LTP (*d*_Br⋯Br_ = 3.68–4.04 Å) indicates the weaker Br⋯Br interactions in 2 (Fig. S14†), such that the smaller [SnBr_6_]^2−^ anion could not anchor the terminal Br atom of the adjacent organic cations to maintain the conformations for organic cations. In short, the complicated and subtle intermolecular interactions between the organic cations and the [SnBr_6_]^2−^ anion essentially brought about the complicated crystal structures for both 2_LTP and 2_HTP. More importantly, it could generate an unusual *cis*-/*anti*-conformational reversal and anomalous ferroelastic phase transitions.

## Conclusions

To summarize, two new polar hybrid ferroelastics, *i.e.*, 1 and 2, were synthesized successfully by the same organic cations (Me_2_NH(CH_2_)_2_Br^+^) but slightly different inorganic octahedra, *i.e.*, relatively larger [TeBr_6_]^2−^ anions in 1 and smaller [SnBr_6_]^2−^ anions in 2. With roughly similar intermolecular interactions, they undergo thermally induced reversible ferroelastic crystalline phase transitions and then solid–liquid phase transitions accompanied by similar and uncommon “low-high-low” SHG switches. However, the slightly different inorganic octahedra endow them with ferroelastic crystalline phase transitions with different symmetry-breaking behaviors, *i.e.*, a normal ferroelastic transition (*P*2_1_ → *Pm*2_1_*n*) resulting from a common order-disorder transition of the well-anchored adjacent organic cations without conformational changes in 1, but an anomalous ferroelastic phase transition (*P*2_1_2_1_2_1_ → *P*2_1_) arising from an unusual *cis*-/*anti*-conformational reversal of organic cations involved in energetically-similar sets of intermolecular interactions in 2. By presenting these two nice instances to understand the anomalous ferroelastic phase transition at the molecular level, this work demonstrates the crucial role of the delicate balance of intermolecular interactions in hybrid crystals in designing nontrivial phase transitions. The information here also provides important clues for the design of new high-temperature ferroelastic multifunctional materials.

## Data availability

All data included in this study are available upon request by contact with the corresponding author.

## Author contributions

W.-X. Zhang and B.-Q Zhao conceived the idea, designed the experiments, and co-wrote the manuscript. B.-Q Zhao performed the synthesis experiments, the DSC, dielectric, crystal structural analyses, ferroelastic domain observations, and the Hirshfeld surface analyses. X.-X. Chen, H. Ye, and Y.-P. Gong assisted the Pawley and Rietveld refinements. J. Wang and L. Ye provided help with the SHG measurements and Hirshfeld surface analyses.

## Conflicts of interest

There are no conflicts to declare.

## Supplementary Material

SC-014-D3SC01101A-s001

SC-014-D3SC01101A-s002

SC-014-D3SC01101A-s003

SC-014-D3SC01101A-s004
