# Supplementary material for: An anomalous ferroelastic phase transition arising from an unusual cis-/anti-conformational reversal of polar organic cations
Source: Chem Sci. 2023 May 2;14(22):5965–73. doi: 10.1039/d3sc01101a (PMC10246694; doi:10.1039/d3sc01101a)
Supplement: SC-014-D3SC01101A-s003 [file SC-014-D3SC01101A-s003.pdf]

Electronic Supplementary Material (ESI) for Chemical Science.
This journal is © The Royal Society of Chemistry 2023

# *Supporting Information*

## An anomalous ferroelastic phase transition arising from an unusual *cis-*/*anti*-conformational reversal of polar organic cations

Bing-Qing Zhao, Xiao-Xian Chen, Hui Ye, Ya-Ping Gong, Jun Wang, Le Ye and Wei-Xiong Zhang*

MOE Key Laboratory of Bioinorganic and Synthetic Chemistry, School of Chemistry and Chemical Engineering, Sun Yat-Sen University, Guangzhou 510275, China.

E-mail: zhangwx6@mail.sysu.edu.cn

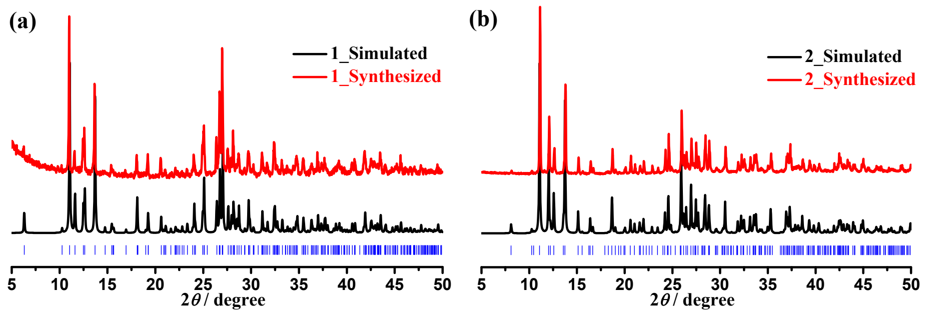


**Fig. S1** Experimental (red) and simulated (black) PXRD patterns for **1** (a) and **2** (b).

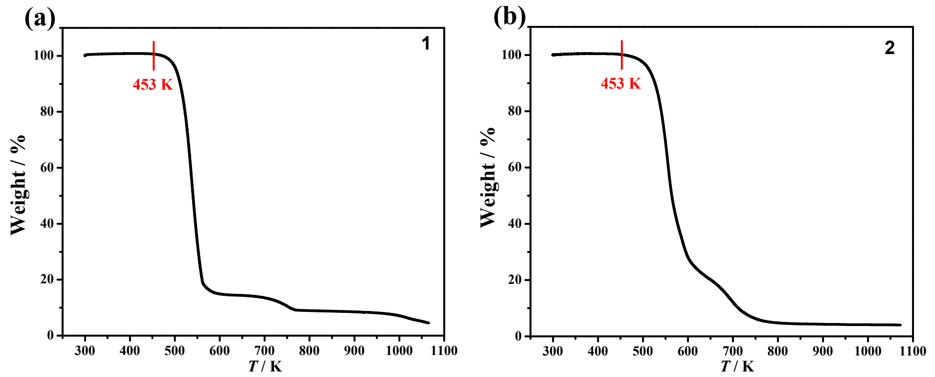


**Fig. S2** Thermogravimetric analyses for **1** (a) and **2** (b).

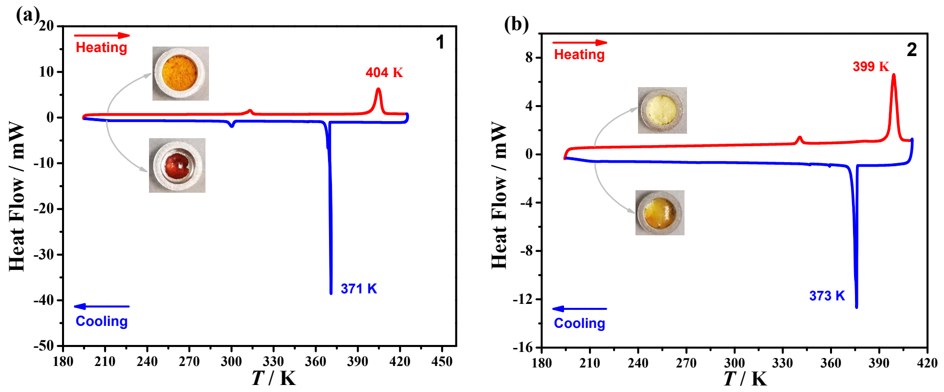


**Fig. S3** DSC curves of melting and solidification for **1** (a) and **2** (b).

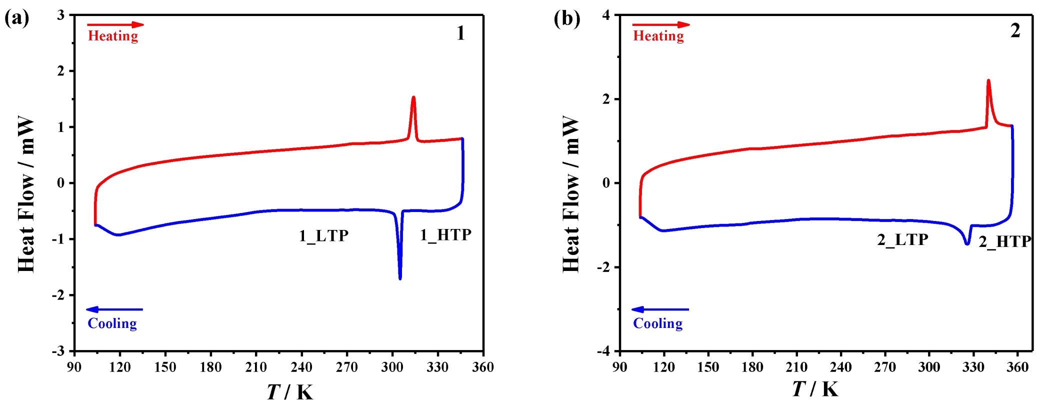


**Fig. S4** DSC curves of **1** (a) and **2** (b) at a temperature as low as 120 K.

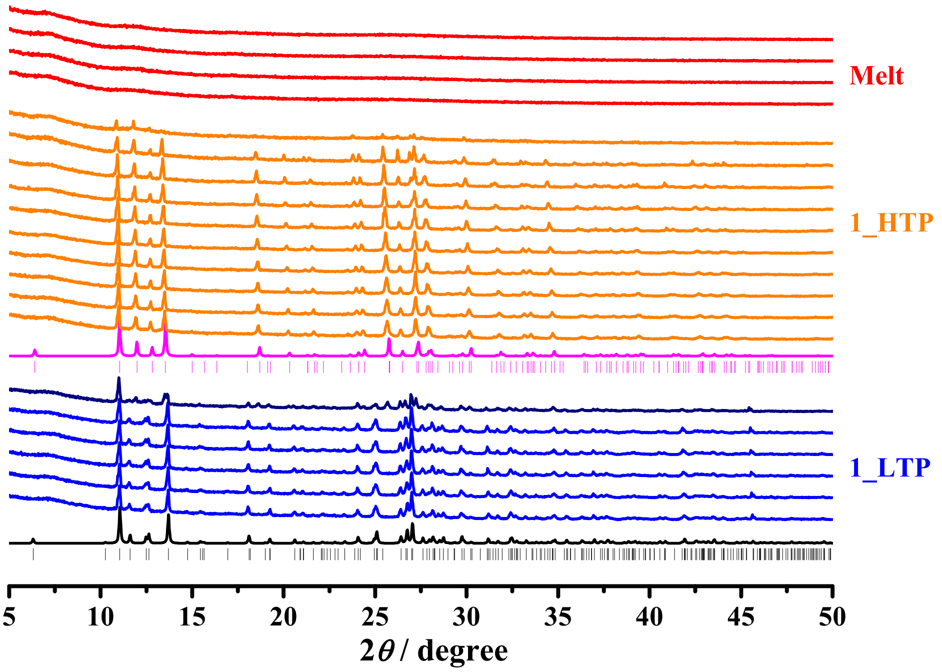


**Fig. S5** The variable-temperature experimental PXRD patterns of **1** and the simulated patterns based on single-crystal structures for **1_LTP** and **1_HTP** phases.

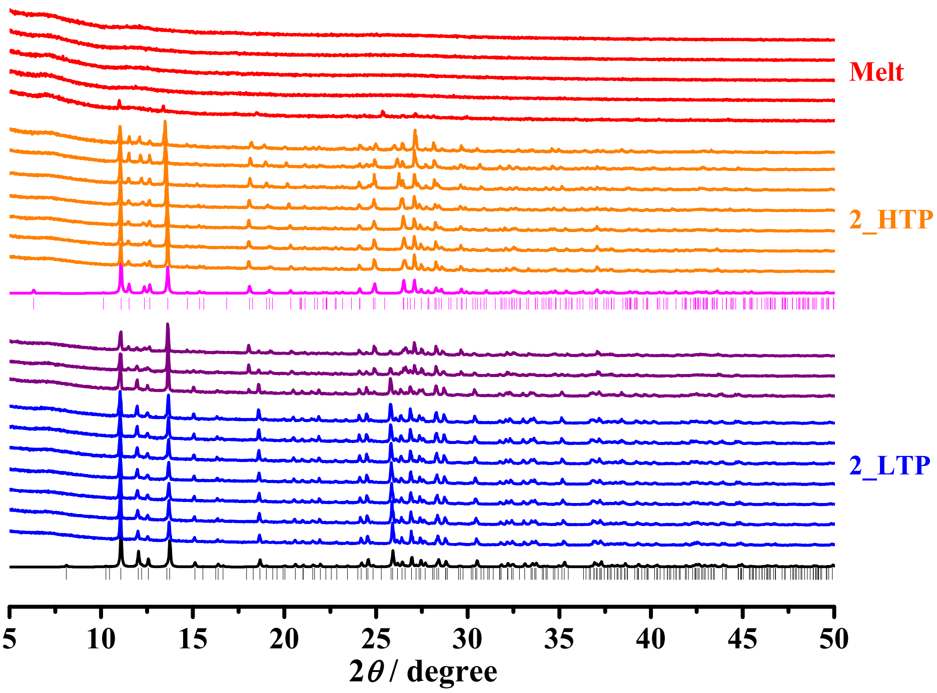


**Fig. S6** The variable-temperature experimental PXRD patterns of **2** and the simulated patterns based on single-crystal structures for **2_LTP** and **2_HTP** phases.

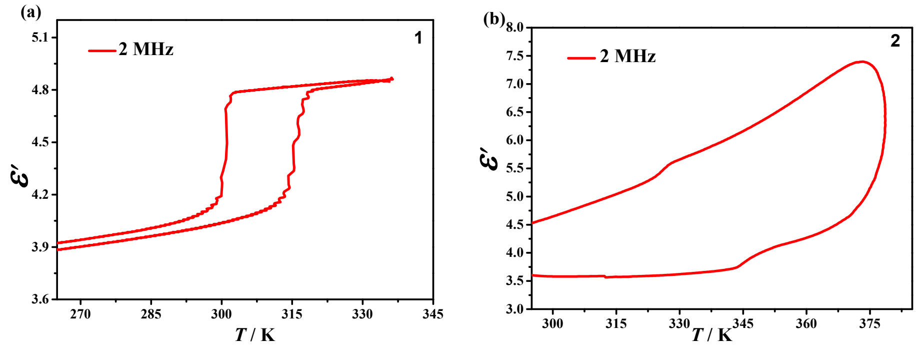


**Fig. S7** The real part of dielectric permittivity of **1** (a) and **2** (b) during a cooling–heating cycle measured under an ac electric field with a frequency of 2 MHz.

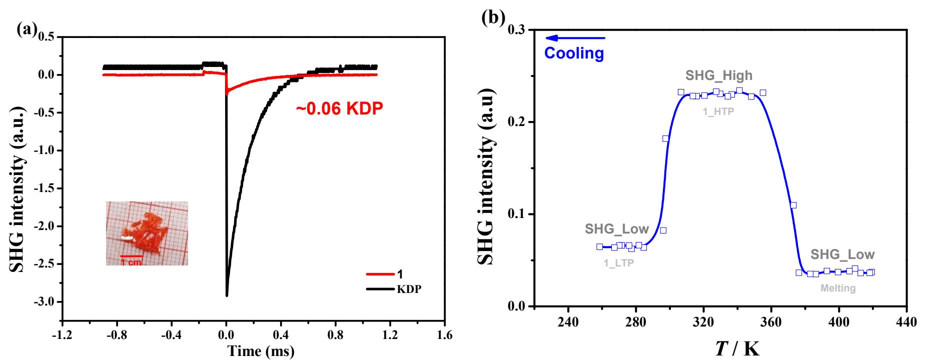


**Fig. S8** (a) Oscilloscope traces of SHG signals of **1** and the referential KDP. (b) The Temperature-dependence of SHG signal in the cooling process after melting for **1.**

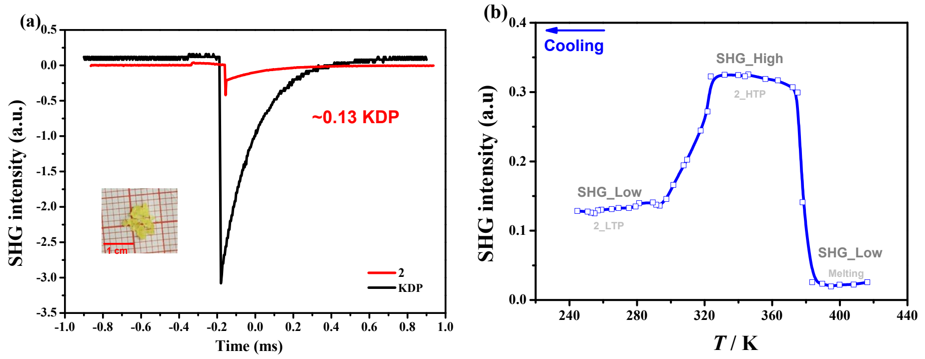


**Fig. S9** (a) Oscilloscope traces of SHG signals of **2** and the referential KDP. (b) The Temperature-dependence of SHG signal in the cooling process after melting for **2.**

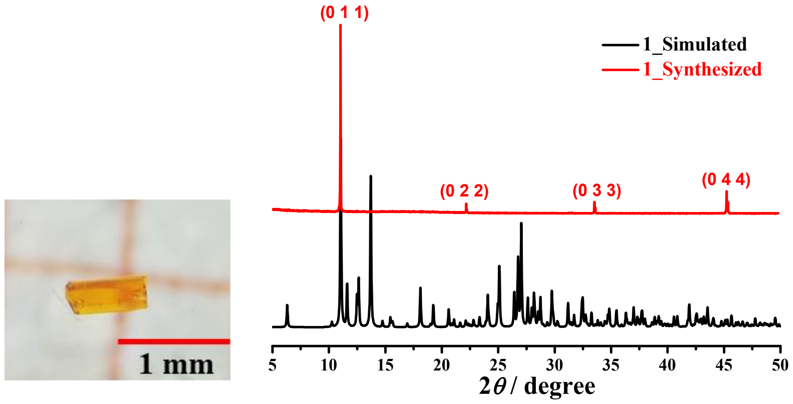


**Fig. S10** The morphology of the single crystal of **1** (left) and X-ray diffraction patterns (right) measured on the largest plane of single crystal for observation of ferroelastic domains.

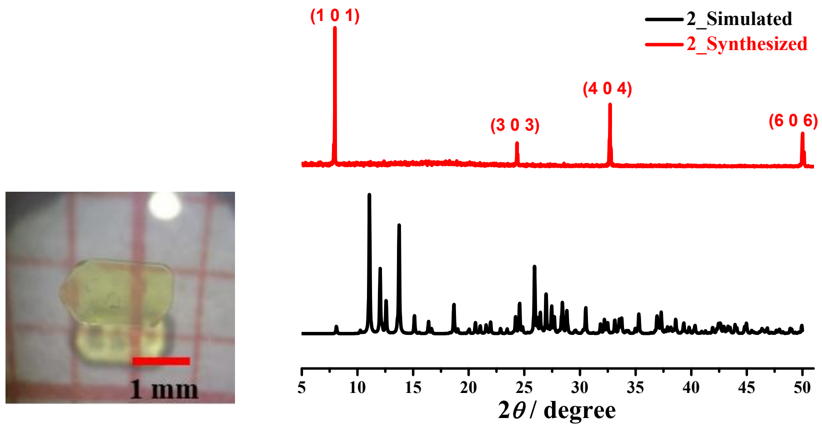


**Fig. S11** The morphology of the single crystal of **2** (left) and X-ray diffraction patterns (right) measured on the largest plane of single crystal for observation of ferroelastic domains.

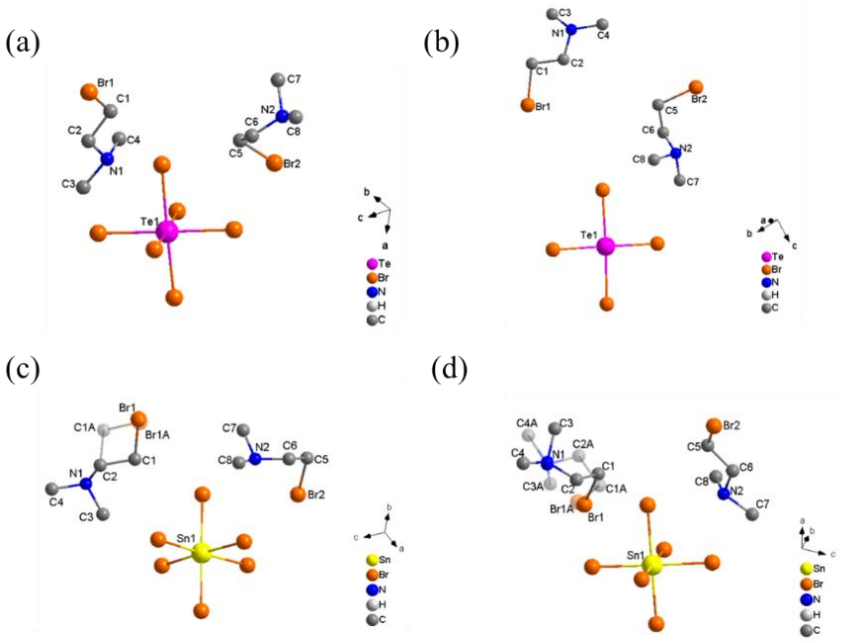


**Fig. S12** The asymmetric units of **1_LTP** (a), **1_HTP** (b), **2_LTP** (c), and **2_HTP** (d).

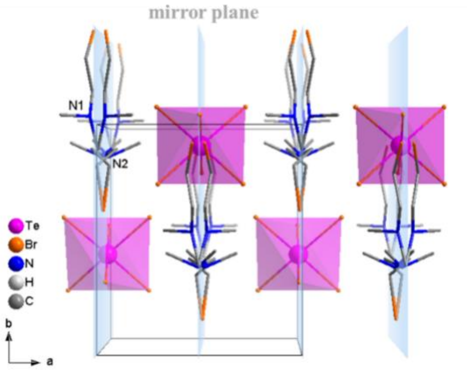


**Fig. S13** The organic cations show 2-fold disorder about the crystallographic mirror plane (shown in the light blue plane) perpendicular to the *a* axis in **1_HTP** phase.

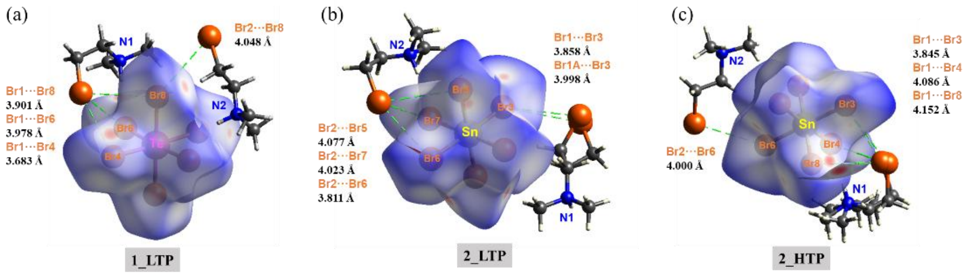


**Fig. S14** Hirshfeld surfaces for the Br···Br contacts and distances between the organic cation inorganic anions in **1_LTP** (a), **2_LTP** (b), and **2_HTP** (c).

**Table S1**. Pawley refinement results on the variable-temperature PXRD patterns of **1**.

| | ***T*/ K** | ***a*/ Å** | ***b*/ Å** | ***c*/ Å** | ***β*/ °** | ***V*/ Å³** | ***R*<sub></sub>P/ %** | ***R*WP/ %** |
|---|---|---|---|---|---|---|---|---|
| **1_LTP** | 273 | 8.602(1) | 9.7260(1) | 14.038(1) | 95.043(2) | 1169.92 | 2.74 | 3.61 |
| | 283 | 8.621(1) | 9.739(1) | 14.056(2) | 94.982(2) | 1175.79 | 2.77 | 3.67 |
| | 293 | 8.639(1) | 9.752(1) | 14.068(1) | 94.874(2) | 1180.92 | 2.74 | 3.62 |
| | 303 | 8.659(1) | 9.766(1) | 14.080(1) | 94.722(3) | 1186.63 | 2.76 | 3.62 |
| | 308 | 8.671(1) | 9.776(1) | 14.086(1) | 94.594(2) | 1190.32 | 2.87 | 3.80 |
| **1_HTP** | 318 | 8.765(3) | 9.858(3) | 13.895(4) | 90 | 1200.60 | 2.87 | 3.81 |
| | 323 | 8.774(1) | 9.869(1) | 13.900(1) | 90 | 1203.66 | 2.86 | 3.91 |
| | 328 | 8.782(1) | 9.876(1) | 13.904(1) | 90 | 1205.91 | 2.72 | 3.74 |
| | 333 | 8.789(1) | 9.881(1) | 13.908(1) | 90 | 1207.88 | 2.77 | 3.81 |
| | 338 | 8.796(1) | 9.888(1) | 13.915(1) | 90 | 1210.29 | 2.81 | 3.72 |

**Table S2**. Pawley refinement results on the variable-temperature PXRD patterns of **2**.

| | ***T*/ K** | ***a*/ Å** | ***b*/ Å** | ***c*/ Å** | ***β*/ °** | ***V*/ Å³** | **$R_P$/ %** | **$R_{WP}$/ %** |
|---|---|---|---|---|---|---|---|---|
| **2_LTP** | 303 | 17.229(1) | 9.687(1) | 14.066(1) | 90 | 2347.66 | 2.90 | 3.85 |
| | 308 | 17.240(1) | 9.690(1) | 14.072(1) | 90 | 2350.89 | 2.88 | 3.85 |
| | 313 | 17.255(1) | 9.694(1) | 14.076(1) | 90 | 2354.71 | 2.82 | 3.72 |
| | 318 | 17.264(1) | 9.697(1) | 14.079(1) | 90 | 2357.00 | 2.70 | 3.55 |
| | 323 | 17.276(1) | 9.700(1) | 14.082(1) | 90 | 2360.05 | 2.70 | 3.55 |
| | 328 | 17.289(1) | 9.703(1) | 14.087(1) | 90 | 2363.35 | 2.78 | 3.66 |
| | 333 | 17.302(1) | 9.707(1) | 14.090(1) | 90 | 2366.54 | 3.31 | 4.53 |
| | 338 | 17.317(1) | 9.710(1) | 14.095(1) | 90 | 2369.89 | 3.04 | 4.15 |
| **2_HTP** | 348 | 8.743(1) | 9.705(1) | 14.025(1) | 94.767(2) | 1185.91 | 4.26 | 6.14 |
| | 353 | 8.753(1) | 9.718(1) | 14.031(1) | 94.681(2) | 1189.47 | 3.48 | 4.80 |
| | 356 | 8.762(1) | 9.726(1) | 14.034(1) | 94.626(2) | 1192.13 | 3.63 | 5.14 |
| | 358 | 8.769(1) | 9.729(1) | 14.038(1) | 94.586(2) | 1193.79 | 3.62 | 5.17 |
| | 361 | 8.778(1) | 9.734(1) | 14.041(1) | 94.507(2) | 1196.02 | 4.13 | 6.10 |
| | 363 | 8.786(1) | 9.7380(2) | 14.043(1) | 94.449(3) | 1197.94 | 4.42 | 6.61 |

**Table S3.** Linear fitting results of the variable-temperature unit-cell parameters of **1.**

| | **Fitting equations** | $R^2$ | **Deduced parameters at $T_1$** |
|---|---|---|---|
| **1_LTP** | *a*/ Å = 0.00194*T* + 8.0707 | 0.9972 | *a* = 8.6798 Å |
| | *b*/ Å = 0.00141*T* + 9.3400 | 0.9936 | *b* = 9.7827 Å |
| | *c*/ Å = 0.00134*T* + 13.6732 | 0.9872 | *c* = 14.0939 Å |
| | *β*/ º = − 0.0126*T* + 98.5248 | 0.9349 | *β* = 94.5652º |
| | *V*/ $Å^3$ = 0.5712*T* + 1013.9270 | 0.9973 | V = 1193.28 $Å^3$ |
| **1_HTP** | *a*/ Å = 0.00156*T* + 8.2700 | 0.9969 | *a* = 8.7599 Å |
| | *c*/ Å = 0.00143*T* + 9.4070 | 0.9722 | *b* = 9.8560 Å |
| | *b*/ Å = 0.00097*T* + 13.5876 | 0.9800 | *c* = 13.8909 Å |
| | | | *β* = 90º |
| | *V*/ $Å^3$ = 0.4720*T* + 1050.8520 | 0.9920 | V = 1199.06 $Å^3$ |

**Table S4.** Linear fitting results of the variable-temperature unit-cell parameters of **2.**

| | **Fitting equations** | $R^2$ | **Deduced parameters at $T_2$** |
|---|---|---|---|
| **2_LTP** | *a*/ Å = 0.00248*T* + 16.4776 | 0.9977 | *a* = 17.3208 Å |
| | *b*/ Å = 0.000643*T* + 9.4926 | 0.9975 | *b* = 9.7112 Å |
| | *c*/ Å = 0.000779*T* + 13.8311 | 0.9941 | *c* = 14.0961 Å |
| | | | *β* = 90º |
| | *V*/ $Å^3$ = 0.6258*T* + 2158.1969 | 0.9983 | V = 2370.96 $Å^3$ |
| **2_HTP** | *a*/ Å = 0.00293*T* + 7.7218 | 0.9835 | *a* = 8.7180 Å |
| | *b*/ Å = 0.00216*T* + 8.9564 | 0.9868 | *b* = 9.6908 Å |
| | *c*/ Å = 0.00123*T* + 13.5958 | 0.9956 | *c* = 14.0140 Å |
| | *β*/ º = − 0.02097*T* + 102.0780 | 0.9834 | *β* = 94.9482º |
| | *V*/ $Å^3$ = 0.8023*T* + 906.5326 | 0.9980 | V = 1179.3044 $Å^3$ |

**Table S5.** Thermal expansion coefficients of the principal axes in **1.**

| | ***T*/ K** | Principle axis | Directions | | | *α*/mk$^{-1}$ | $β_V$/mk$^{-1}$ |
|---|---|---|---|---|---|---|---|
| | | | *a*/ Å | *b*/ Å | *c*/ Å | | |
| **1_LTP** | 273-308 | $X_1$ | 0.6112 | -0.0000 | -0.7914 | 41(11) | 488(8) |
| | | $X_2$ | 0.0000 | -1.0000 | -0.0000 | 144(4) | |
| | | $X_3$ | 0.9496 | -0.0000 | 0.3135 | 299(12) | |
| **1_HTP** | 318-338 | $X_1$ | 0.0000 | 0.0000 | 1.0000 | 69(3) | 394(12) |
| | | $X_2$ | 0.0000 | 1.0000 | -0.0000 | 147(7) | |
| | | $X_3$ | -1.0000 | 0.0000 | 0.0000 | 174(6) | |

**Table S6.** Thermal expansion coefficients of the principal axes in **2.**

| | *T*/ K | Principle axis | Directions | | | *α*/MK$^{-1}$ | $β_V$/MK$^{-1}$ |
|---|---|---|---|---|---|---|---|
| | | | *a*/ Å | *b*/ Å | *c*/ Å | | |
| **2_LTP** | 303-338 | $X_1$ | 0.0000 | 0.0000 | 1.0000 | 56(1) | 267(2) |
| | | $X_2$ | 0.0000 | 1.0000 | -0.0000 | 66(1) | |
| | | $X_3$ | -1.0000 | 0.0000 | 0.0000 | 144(2) | |
| **2_HTP** | 348-363 | $X_1$ | 0.6296 | -0.0000 | -0.7769 | 4(3) | 678(13) |
| | | $X_2$ | 0.0000 | -1.0000 | 0.0000 | 222(9) | |
| | | $X_3$ | 0.9433 | -0.0000 | 0.3320 | 448(23) | |

**Table S7.** Selected bond lengths (Å) and bond angles (º) for **1** at **LTP** and **HTP**.

| | | | | |
|---|---|---|---|---|
| **1_LTP** | Te1-Br3 | 2.721(3) | Te1-Br6 | 2.689(2) |
| | Te1-Br4 | 2.670(2) | Te1-Br7 | 2.736(2) |
| | Te1-Br5 | 2.679(2) | Te1-Br8 | 2.695(2) |
| | ∠Br3-Te1-Br7 | 90.49(8) | ∠Br5-Te1-Br7 | 92.22(7) |
| | ∠Br4-Te1-Br3 | 87.24(8) | ∠Br5-Te1-Br8 | 89.89(7) |
| | ∠Br4-Te1-Br5 | 90.01(7) | ∠Br6-Te1-Br3 | 92.44(9) |
| | ∠Br4-Te1-Br6 | 91.21(8) | ∠Br6-Te1-Br7 | 90.81(8) |
| | ∠Br4-Te1-Br7 | 177.02(8) | ∠Br6-Te1-Br8 | 177.25(9) |
| | ∠Br4-Te1-Br8 | 91.21(7) | ∠Br8-Te1-Br3 | 88.98(8) |
| | ∠Br5-Te1-Br3 | 177.01(9) | ∠Br8-Te1-Br7 | 86.82(7) |
| | ∠Br5-Te1-Br6 | 88.80(8) | | |
| **1_HTP** | Te1-Br3 | 2.664(5) | Te1-Br5 | 2.724(7) |
| | Te1-Br4 | 2.734(3) | Te1-Br6 | 2.647(4) |
| | ∠Br3-Te1-Br4 | 89.63(10) | ∠Br6-Te1-Br4A | 90.10(15) |
| | ∠Br3-Te1-Br5 | 177.7(2) | ∠Br6-Te1-Br4 | 178.84(16) |
| | ∠Br4-Te1-Br4A | 90.99(15) | ∠Br6-Te1-Br5 | 90.86(19) |
| | ∠Br5-Te1-Br4 | 88.77(14) | ∠Br6-Te1-Br6A | 88.8(3) |
| | ∠Br6-Te1-Br3 | 90.77(16) | | |
| | A) 1−x, +y, +z | | | |

**Table S8.** Selected bond lengths (Å) and bond angles (º) for **2** at **LTP** and **HTP**.

| | | | | |
|---|---|---|---|---|
| **2_LTP** | Sn1-Br3 | 2.617(1) | Sn1-Br6 | 2.586(1) |
| | Sn1-Br4 | 2.600(1) | Sn1-Br7 | 2.596(1) |
| | Sn1-Br5 | 2.581(1) | Sn1-Br8 | 2.619(1) |
| | ∠Br3-Sn1-Br8 | 87.79(4) | ∠Br5-Sn1-Br8 | 176.96(5) |
| | ∠Br4-Sn1-Br3 | 90.35(4) | ∠Br6-Sn1-Br3 | 177.22(5) |
| | ∠Br4-Sn1-Br8 | 90.37(3) | ∠Br6-Sn1-Br4 | 88.76(4) |
| | ∠Br5-Sn1-Br3 | 89.30(4) | ∠Br6-Sn1-Br7 | 90.44(5) |
| | ∠Br5-Sn1-Br4 | 90.53(4) | ∠Br6-Sn1-Br8 | 89.58(4) |
| | ∠Br5-Sn1-Br6 | 93.34(5) | ∠Br7-Sn1-Br3 | 90.38(5) |
| | ∠Br5-Sn1-Br7 | 90.87(4) | ∠Br7-Sn1-Br4 | 178.43(4) |
| | ∠Br7-Sn1-Br8 | 88.26(4) | | |
| **2_HTP** | Sn1-Br3 | 2.597(4) | Sn1-Br6 | 2.613(4) |
| | Sn1-Br4 | 2.601(4) | Sn1-Br7 | 2.604(4) |
| | Sn1-Br5 | 2.587(4) | Sn1-Br8 | 2.604(4) |
| | ∠Br3-Sn1-Br4 | 89.97(12) | ∠Br5-Sn1-Br3 | 89.65(14) |
| | ∠Br3-Sn1-Br6 | 178.01(17) | ∠Br5-Sn1-Br4 | 91.13(13) |
| | ∠Br3-Sn1-Br7 | 91.49(14) | ∠Br5-Sn1-Br6 | 91.46(17) |
| | ∠Br3-Sn1-Br8 | 89.57(13) | ∠Br5-Sn1-Br7 | 89.89(15) |
| | ∠Br4-Sn1-Br6 | 88.36(15) | ∠Br5-Sn1-Br8 | 177.70(16) |
| | ∠Br4-Sn1-Br7 | 178.23(17) | ∠Br7-Sn1-Br6 | 90.17(17) |
| | ∠Br4-Sn1-Br8 | 91.03(13) | ∠Br7-Sn1-Br8 | 87.97(14) |
| | ∠Br8-Sn1-Br6 | 89.39(16) | | |

**Table S9.** Calculated polarization intensity along the *b* axis for **1** and **2** by point charge model.[S1, S2]

| Compound | Polarization Intensity ($C/m^2$) |
|---|---|
| **1_LTP** | $3.17\times10^{-3}$ |
| **1_HTP** | $-8.95\times10^{-2}$ |
| **2_LTP** | $-3.15\times10^{-2}$ |
| **2_HTP** | $4.40\times10^{-1}$ |

**The equations for calculation of the spontaneous strain.**

The spontaneous strain tensors can be given as: [S3, S4]

For *mm*2F2:

$$\varepsilon_{ij} = \begin{bmatrix} \frac{a_{1\_LTP}}{a_{1\_HTP}} - 1 & 0 & \frac{1}{2}\left(\frac{c_{1\_LTP}}{c_{1\_HTP}}\cos\beta\right) \\ 0 & \frac{b_{1\_LTP}}{b_{1\_HTP}} - 1 & 0 \\ \frac{1}{2}\left(\frac{c_{1\_LTP}}{c_{1\_HTP}}\cos\beta\right) & 0 & \frac{c_{1\_LTP}}{c_{1\_HTP}}\sin\beta - 1 \end{bmatrix} = 0.0594$$

For 222F2:

$$\varepsilon_{ij} = \begin{bmatrix} \frac{2a_{2\_HTP}}{a_{2\_LTP}} - 1 & 0 & \frac{1}{2}\left(\frac{c_{2\_HTP}}{c_{2\_LTP}}\cos\beta\right) \\ 0 & \frac{b_{2\_HTP}}{b_{2\_LTP}} - 1 & 0 \\ \frac{1}{2}\left(\frac{c_{2\_HTP}}{c_{2\_LTP}}\cos\beta\right) & 0 & \frac{c_{2\_HTP}}{c_{2\_LTP}}\sin\beta - 1 \end{bmatrix} = 0.0618$$

**References:**

(S1) F. Jensen, *Phys. Chem. Chem. Phys.*, 2022, **24**, 1926-1943.

(S2) G. G. Hall, *Chem. Phys. Lett.*, 1973, **20**, 501-503.

(S3) K. Aizu, *J. Phys. Soc. Jpn.*, 1970, **28**, 706-716.

(S4) J. Sapriel, *Phys. Rev. B*, 1975, **12**, 5128-5140.
